# Myofascial release and fascial-targeted mechanical interventions in musculoskeletal rehabilitation: mechanisms, modalities, and integrative physiology

**DOI:** 10.3389/fphys.2026.1801306

**Published:** 2026-03-27

**Authors:** Yuqing Gao, Dongxu Gao

**Affiliations:** 1 School of Physical Education, Wuhan Sports University, Wuhan, China; 2 Division of Sports Science and Physical Education, Tsinghua University, Beijing, China

**Keywords:** fascia, fascial-targeted mechanical interventions, instrument-assisted soft tissue mobilization, musculoskeletal rehabilitation, myofascial pain, myofascial release

## Abstract

Fascia is a continuous connective tissue network that surrounds and integrates muscles, bones, nerves, and organs, contributing to force transmission, postural organization, movement coordination, and sensory processing within the musculoskeletal system. Any alterations in fascial properties, including increased stiffness, adhesions, and densification, have been associated with pain, restricted mobility, and functional impairment in musculoskeletal conditions. These associations have supported the growing use of myofascial release interventions within rehabilitation practice. Mechanical loading of fascial tissues through pressure, shear, vibration, or acoustic stimulation may influence tissue mechanics and sensory signaling. Across manual, instrument-assisted, and device-based modalities, myofascial release interventions apply these mechanical stimuli through different modes while sharing overlapping physiological targets. Proposed mechanisms include modulation of tissue mechanics, sensory receptor stimulation, and neurophysiological effects on muscle tone and pain perception. Evidence reported in the literature indicates that myofascial release interventions are frequently associated with short-term improvements in pain and joint range of motion. In contrast, findings related to long-term functional outcomes and direct, modality-specific structural adaptation of fascial tissues remain inconsistent. Interpretation of available data is further constrained by heterogeneity in intervention protocols, operator dependence, variable outcome measures, and limited use of objective methods capable of quantifying fascial mechanical properties *in vivo*. Within an integrative physiological framework, myofascial release interventions are most consistently supported as adjunctive components of musculoskeletal rehabilitation rather than stand-alone treatments. Their clinical value appears greatest when used to facilitate movement, reduce symptom burden, and enhance engagement with active rehabilitation strategies such as exercise and movement re-education. Continued advancement in this field will depend on standardized reporting, improved methodological rigor, longer-term follow-up, and the incorporation of objective assessments to clarify mechanisms and guide evidence-based integration.

## 1 Introduction

Fascia is a continuous three-dimensional connective tissue matrix that surrounds and interweaves all muscles, bones, nerves, and organs of the body. Beyond its ability to act as an inert packing material, fascia is recognized as a dynamic tissue with important roles. It plays a significant role in force transmission, postural support, and sensory feedback (Stecco et al., 2025). There exists a dense network of mechanoreceptors and nociceptors within fascial tissues that contribute to proprioception and pain perception (Suarez-Rodriguez et al., 2022). When healthy, fascia is pliable and allows muscles and structures to glide smoothly. However, in dysfunction, fascia can become stiff, adherent, or thickened. It can lead to pain and restricted mobility (Stecco et al., 2025). Fascial dysfunction has been implicated in a variety of musculoskeletal conditions, such as low back pain and myofascial pain syndrome. In myofascial pain syndrome, abnormalities such as fascial fibrosis, densification (excessive collagen or hyaluronan accumulation), and adhesions between layers may generate nociceptive input and limit movement. These perceptions have driven the developing interest in therapies that specifically target fascia (Gromakovskis, 2025).

**Figure 1:**
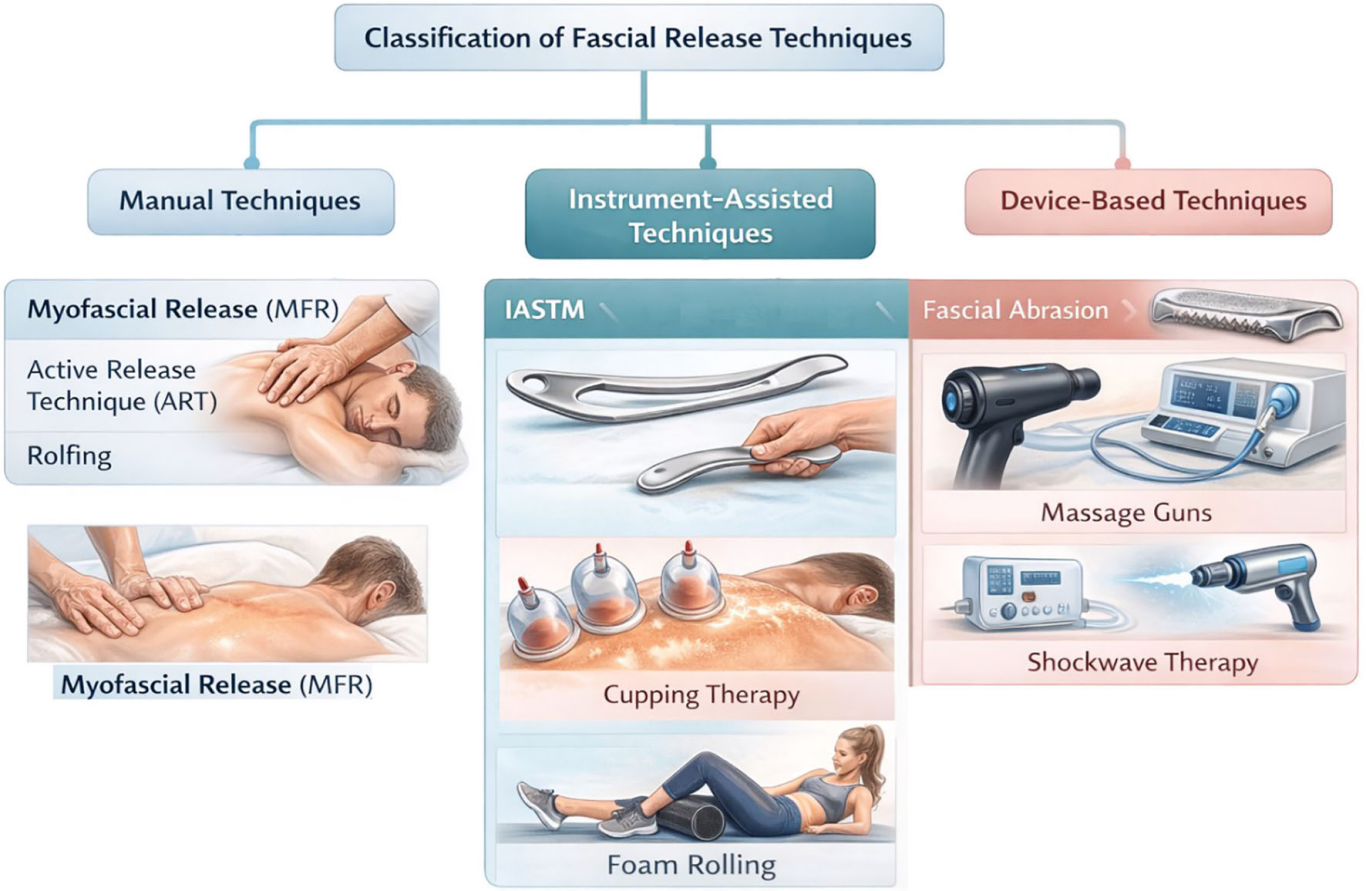
Schematic classification of myofascial release and fascial-targeted mechanical interventions. Primary myofascial release modalities include manual and instrument-assisted techniques, while selected device-based modalities are classified as secondary fascial-targeted mechanical interventions based on their mechanisms and clinical applications. The conceptual diagram illustrates three major categories: manual techniques (e.g., myofascial release, active release technique, and structural integration), instrument-assisted techniques (e.g., instrument-assisted soft tissue mobilization, cupping therapy, and foam rolling), and device-based techniques (e.g., percussive massage devices and extracorporeal shockwave therapy). The figure is intended as an illustrative overview of modality classification rather than a depiction of specific clinical protocols.

Myofascial release and fascial-targeted mechanical interventions refer to interventions which are aimed at restoring normal mobility and extensibility of fascial tissues. In rehabilitation medicine, clinicians employ a spectrum of such techniques. These techniques range from hands-on manual therapies to tool-assisted methods and modern device-based modalities. Myofascial release techniques can be broadly classified into primary manual and instrument-assisted techniques, while selected device-based modalities may be considered secondary fascial-targeted mechanical interventions depending on their mechanism and clinical application, as illustrated in **Figure 1**. Every approach has a different mode of application but shares the common goal of releasing fascial tension, breaking down adhesions, and reducing pain (Antohe et al., 2024). Manual fascial release (MFR) typically involves the hands of a therapist who applies sustained pressure, traction, or stretch to the fascia of the patient until release is felt. On the other hand, Instrument-assisted soft tissue mobilization (IASTM) uses rigid tools to scrape or compress tissue, ostensibly to detect and treat adhesions/scar tissue. Likewise, device-based approaches use both low-tech tools used by patients themselves (like foam rollers or massage balls for self-myofascial release) and high-tech therapeutic devices (such as vibration massage guns and extracorporeal shockwave machines). Both these tools aim to mobilize fascia through mechanical forces. Despite their widespread use, the relative efficacy & ideal applications of these techniques remain areas of active investigation and debate (Sur et al., 2024).

In this review, myofascial release (MFR) is defined as a group of mechanically applied interventions specifically which are intended to modify the mechanical behavior, mobility, or sensory function of fascial tissues and the myofascial system. Primary myofascial release modalities include manual myofascial release and instrument-assisted soft tissue mobilization (IASTM). These directly apply sustained compression, shear, or traction to fascial layers. Secondary fascial-targeted mechanical interventions, including self-myofascial release tools (e.g., foam rollers) and selected device-based modalities (e.g., percussive therapy and extracorporeal shockwave therapy), are included when their therapeutic intent, clinical application, or proposed mechanisms involve modulation of fascial tissue behavior, fascial mechanoreceptors, or fascial-related pain and mobility dysfunction. Interventions whose primary mechanism does not directly involve mechanical interaction with fascial tissues are discussed only where relevant to fascial physiology or myofascial pain mechanisms. This distinction allows clarification between true myofascial release techniques and broader soft tissue interventions that may secondarily influence fascial function.

Against this background, a comprehensive integrative perspective on myofascial release interventions is warranted. The present narrative synthesis brings together current concepts of fascial anatomy and physiology with available evidence on mechanical and neurophysiological bases of commonly applied fascial release approaches in musculoskeletal rehabilitation. By integration of findings across manual, instrument-assisted, and device-mediated modalities, this work contextualizes proposed mechanisms, summarizes reported clinical effects on pain and movement, and highlights methodological limitations and knowledge gaps that constrain interpretation. Such an integrative framework aims to support physiologically grounded clinical reasoning and to inform future research directions focused on objective assessment and evidence-based integration of fascial release within active rehabilitation strategies. Furthermore, interventions whose primary mechanism does not involve direct or indirect mechanical interaction with fascial tissues are outside the primary scope of this review and are discussed only where relevant to fascial physiology or myofascial pain mechanisms.

## 2 Structural organization and physiological functions of the fascial system

**Figure 2:**
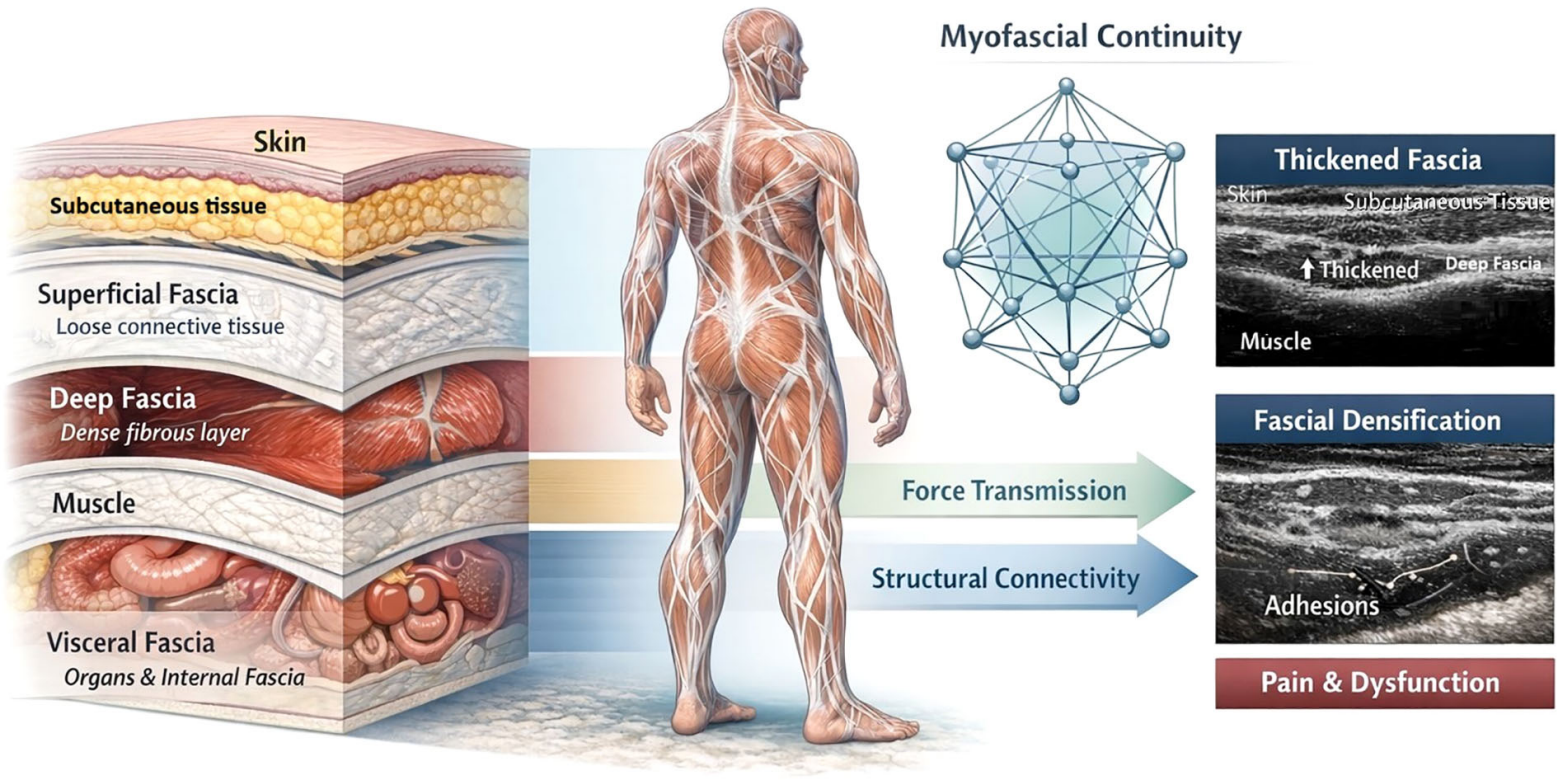
Conceptual schematic illustrating the structural organization and functional continuity of the fascial system. The schematic depicts layered fascial architecture including skin, subcutaneous tissue (superficial fascia), deep fascia, and visceral fascia, highlighting global myofascial connectivity and force transmission. Representative ultrasound images illustrate thickening of the deep fascia (hyperechoic band between subcutaneous tissue and muscle) and fascial densification/adhesion-related changes, which may be associated with pain and movement dysfunction.

Fascia is a ubiquitous connective tissue which forms continuous web throughout body. It consists primarily of collagen (providing tensile strength) and elastin fibers which are embedded in a hydrated ground substance rich in glycosaminoglycans. Anatomically, fascia is often described in layers. Superficial fascia resides just beneath the skin and is composed of loose areolar connective tissue with variable content of fat. It helps to facilitate mobility of skin and houses nerves and blood vessels (Fede et al., 2025). Deep fascia is a tough and dense connective tissue layer that compartmentalizes and invests muscles & organs. There are also visceral fasciae that suspend and separate internal organs (Boser and Schumaker, 2024). All these fascial components are interconnected. The fascial system builds a three-dimensional continuum through the body. The tension in one region can be transmitted to distant areas, which reflects a tensegrity-like biomechanical model (Bordoni and Escher, 2025). The continuous three-dimensional organization and functional connectivity of the fascial system are illustrated in **Figure 2**.

Physiologically, fascia plays multiple roles. It provides structural support and alignment for muscles and joints. It acts as a passive harness that maintains posture and coordinates the actions of muscles. Fascial tissues also store & release elastic energy (Fede et al., 2025). Importantly, fascia is richly innervated. It contains abundant sensory receptors such as mechanoreceptors (e.g., Ruffini and Pacini corpuscles) and free nerve endings that serve as nociceptors. This extensive innervation means that the fascia is a critical organ of proprioception and pain. In normal movement, fascia ensures smooth gliding of muscle fibers and distributes mechanical loads. However, when fascia loses its flexibility or exhibits adhesions, it can limit the range of motion and alter muscle movement (Sezik and Sezik, 2023). Fascial adhesions often occur after trauma, surgery, or prolonged immobilization. Likewise, fascial densification arises from changes in the hyaluronan content or viscosity in the extracellular matrix. This causes an increase in tissue stiffness and impaired sliding of fascial planes (Pratt, 2021).

Clinically, compromised fascial mechanics have been linked to various syndromes. Chronic low back pain patients have increased thickness and stiffness of the thoracolumbar fascia on ultrasound imaging as compared to healthy controls (Langevin et al., 2009). Fibromyalgia (a diffuse pain condition) has also been hypothesized to involve global fascial abnormalities or central nervous system processing of fascial sensory input. Due to the broad impact of fascia on musculoskeletal function and pain, interventions that normalize fascial properties are biologically plausible treatments (Siracusa et al., 2021). Enhancement of fascial glide, breakage of restrictive cross-links, and restoration of normal viscoelasticity may help reduce pain and improve mobility. Additionally, stimulation of fascial mechanoreceptors can induce neurological modulation. For instance, slow, sustained fascial stretches are thought to trigger a parasympathetic response and decrease muscle tone via spinal reflex pathways. These concepts form the rationale behind various myofascial release and fascial-targeted mechanical interventions (Ide et al., 2026).

## 3 Manual myofascial release interventions: mechanical and neurophysiological modulation

Manual fascial release techniques are administered directly by a therapist’s hands (or occasionally elbows or forearms). It is done to manipulate the fascial tissues of the patient. These techniques date back decades and encompass a range of approaches and schools of thought. Unlike classic muscle massage, which often involves rhythmic stroking or kneading, myofascial release (MFR) typically involves sustained pressure or stretches. This pressure is held for extended periods (e.g., 90 seconds or longer) to allow the fascia to elongate and melt. The pressure is usually gentle to moderate and applied slowly (Shults and Fritsch, 2025).

### 3.1 Modes of manual fascial loading and tissue interaction

One of the widely known approaches is the John F. Barnes’ Myofascial Release method. It emphasizes sustained, gentle pressure on fascial restrictions until softening is felt. The Barnes’ approach and similar indirect MFR techniques rely on concept of fascia as continuous web. The release in one area can affect distant regions. Those therapists who are trained in this method often describe waiting for creep in tissue and use very little lubricant to maintain traction on fascia rather than gliding over skin (Barnes and Rivera, 2022). In contrast, direct myofascial release or deep manual therapy techniques (such as Rolfing^®^ Structural Integration) apply more firm pressure or even strong elbow pressure to break adhesions and remodel tissue (Brandl et al., 2022). Rolfing Structural Integration was developed by Ida Rolf. It is systematic program of ten sessions that aims to rebalance entire fascia of the body. Rolfers use knuckles, fists, and elbows to deliver deep and slow strokes along fascial planes and attachments. Some evidence suggests that structural integration can enhance range of motion and posture maintenance. Although overall research on Rolfing’s efficacy is limited and mixed (Bohunicky et al., 2024).

Another manual technique is the Active Release Technique (ART). It is commonly used by sports chiropractors and physical therapists. ART combines manual pressure on a muscle-fascia unit with active or passive movement of the limb to stretch tissue under tension (Barnes and Rivera, 2022). In this technique, the therapist pin down a tight band in calf and then dorsiflex the ankle to elongate the tissue beneath the contact point. It breaks adhesions and restores sliding between muscle & fascia. Trigger point release can also be considered a manual fascial technique. In this technique, the therapist palpates for hyperirritable nodules (trigger points) and applies ischemic compression until the point softens. The release of trigger points may decompress sensory nerves and improve local fascial pliability. It thus reduces pain referral (Landfald et al., 2025).

### 3.2 Biomechanical, neurophysiological, and cellular mechanisms

**Figure 3:**
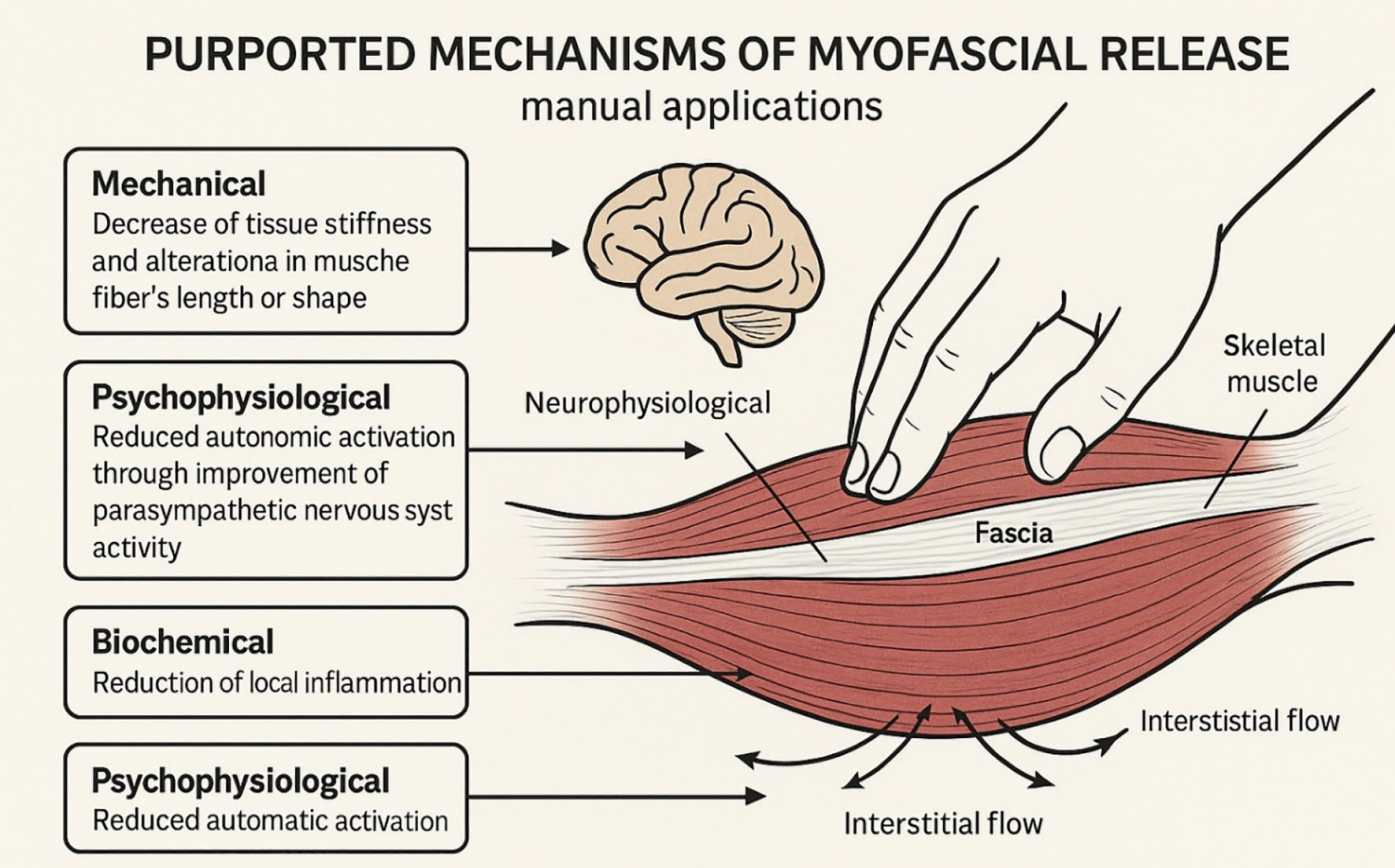
Myofascial release may exert therapeutic effects through multiple interacting mechanical, neurophysiological, and biochemical pathways. Mechanical effects include reductions in tissue stiffness, fascial creep, and improved inter-layer glide resulting from viscoelastic deformation and structural remodeling (Ide et al., 2026). Neurophysiological and psychophysiological mechanisms involve stimulation of fascial mechanoreceptors, modulation of nociceptive signaling, and reductions in sympathetic nervous system activity, contributing to decreased muscle tone and pain perception (Khan et al., 2022; Takenaka et al., 2023). Improvements in circulation and interstitial fluid dynamics may facilitate removal of inflammatory mediators and metabolic by-products, supporting tissue recovery and pain reduction. In addition, inflammatory remodeling, fibroblast activation, and extracellular matrix adaptation may contribute to longer-term structural and functional improvements (Ide et al., 2026).

The proposed mechanisms of manual fascial release are multifaceted. It involves both mechanical and neurological effects. **Figure 3** illustrates the proposed mechanical, biochemical, and neurophysiological mechanisms underlying the effects of Myofascial release and fascial-targeted mechanical interventions. Mechanically, sustained pressure or stretch may deform collagen and the extracellular matrix. It breaks apart aberrant cross-links between fibers and restores extensibility (Forman and Mela, 2025). Fascial creep during a long hold can also result in thixotropy, which could improve the sliding of the fascial layer (Kodama et al., 2023). Additionally, manual tension may stimulate local fibroblasts to remodel tissue.

**Figure 4:**
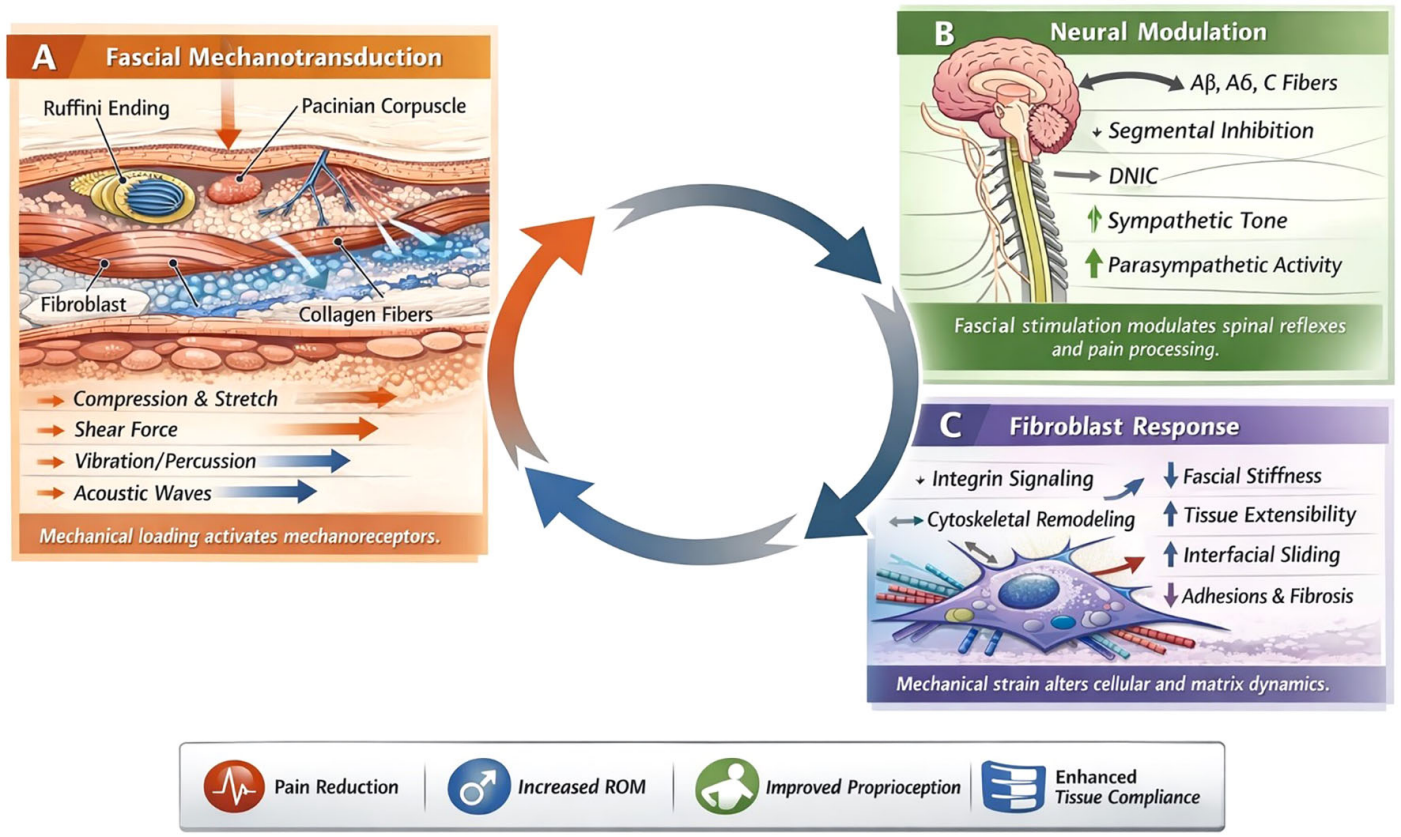
Schematic conceptual model of biomechanical, neurophysiological, and cellular mechanisms underlying myofascial release interventions. **(A)** Fascial mechanotransduction: Mechanical loading through manual, instrument-assisted, and device-based techniques (compression, stretch, shear, vibration, and acoustic stimulation) deforms the extracellular matrix, activates mechanoreceptors (e.g., Ruffini and Pacinian corpuscles), and initiates fibroblast-mediated mechanotransduction processes. **(B)** Neural modulation: Afferent sensory input from fascial mechanoreceptors influences spinal and supraspinal pathways, contributing to segmental inhibition, altered motor neuron excitability, and modulation of sympathetic and parasympathetic nervous system activity involved in pain processing and muscle tone regulation. **(C)** Fibroblast and extracellular matrix response: Mechanical forces stimulate integrin signaling, cytoskeletal remodeling, and extracellular matrix adaptation, contributing to changes in fascial stiffness, interfacial sliding, and tissue extensibility.

On the neurophysiological aspect, manual pressure stimulates cutaneous and fascial mechanoreceptors, including Ruffini endings and interstitial receptors, which modulate muscle tone through spinal reflex pathways and supraspinal influences while also reducing sympathetic nervous system activity and enhancing parasympathetic regulation (Khan et al., 2022; Takenaka et al., 2023). This autonomic modulation may contribute to reduced muscle tension, decreased nociceptive sensitivity, and improved neuromuscular control, while also influencing local vascular perfusion and tissue oxygenation. Thus, after MFR, patients often feel not only an increased range of motion but also generalized relaxation. It triggers a reduction in sympathetic nervous system activity and alters pain signaling pathways (akin to a therapeutic neuromodulation) (Khan et al., 2022). MFR can decrease the excitability of motor neurons and reduce EMG activity in resting muscle and support centrally mediated muscle relaxation effects (Takenaka et al., 2023). Furthermore, by improving circulation (blood and lymphatic flow) in the treated area, manual release may help wash out pro-inflammatory cytokines and metabolic byproducts that contribute to pain and tissue dysfunction, thereby contributing to reduction of local inflammation and improved tissue recovery. These mechanical, neurophysiological, and biochemical mechanisms are closely interconnected, as mechanical stimulation initiates neural responses that influence autonomic regulation, circulation, inflammatory processes, and cellular remodeling (Wiewelhove et al., 2019; Cheatham et al., 2015). The integrated biomechanical, neurophysiological, and cellular mechanisms underlying different fascial release modalities are summarized in **Table 1** and **Figure 4**.

**Table 1:** Biomechanical, neurophysiological, and cellular mechanisms underlying different fascial release modalities.

Intervention category	Nature of tissue interaction	Primary biomechanical mechanisms	Neuro-physiological pathways	Cellular/tissue-level responses	References
Manual Fascial Release (MFR)	Direct manual deformation with neuromodulatory effects	Low-load stretch, fascial creep, reduced friction	Ruffini stimulation, parasympathetic activation, spinal inhibition	Altered fibroblast activity, improved glide, reduced stiffness	(Ide et al., 2026)
Direct Deep Fascial Techniques	Mechanical remodeling of dense fascial layers	High-pressure shear, adhesion disruption	Nociceptive input with descending modulation	Collagen realignment, fibrosis breakdown	(Shamsi et al., 2024; Ide et al., 2026)
Active Fascial Techniques (ART)	Movement-based restoration of sliding	Compression with elongation	Proprioceptive recalibration	Improved muscle–fascia interface mobility	(Barnes and Rivera, 2022)
IASTM	Localized micro-disruption	Transverse friction, micro-trauma	Increased afferent input	Inflammatory remodeling, neovascularization	(MacDonald et al., 2016; Rowlett et al., 2019; Lin et al., 2025)
Fascial Decompression (Cupping)	Indirect separation, vascular stimulation	Tissue lifting, fluid redistribution	Cutaneous mechanoreceptor stimulation	Improved perfusion, fascial layer separation	(Mohamed et al., 2023; Jia et al., 2025)
Self-Myofascial Release (SMR)	Indirect mechanical pressure	Repetitive rolling shear	DNIC, central pain modulation	Transient viscosity change, increased circulation	(Cheatham et al., 2015; Ferreira et al., 2022; Martínez-Aranda et al., 2024)
Percussive/Vibration Devices	Mechanical stimulation, sensory analgesia	High-frequency oscillation	Vibration analgesia, spindle modulation	Reduced tone, temporary compliance	(Sams et al., 2023)
Shockwave Therapy (ESWT)	Deep biological stimulation	Acoustic mechanical loading	Substance P reduction, desensitization	Neovascularization, tissue regeneration	(Ryskalin et al., 2022; De la Corte-Rodríguez et al., 2023; Avendaño-López et al., 2024)

### 3.3 Contextual application and methodological constraints

In practice, manual fascial release is highly operator-dependent. The effectiveness can vary with the skill, experience, and sensitivity of the therapist. Clinicians often palpate for subtle tissue texture changes, temperature differences, or tight lines of pull in fascia to target their intervention. Sessions may last from a few minutes on a focal area to an hour for full-body integrative treatment (Manheim, 2024). One advantage of manual techniques is the direct feedback that the therapist receives through their hands. They can adjust pressure and direction in real-time as tissue responds. There are minimal equipment needs (just a treatment table and some lubricant for certain techniques). It makes it a readily accessible modality. Patients often report subjective improvement in pain and mobility after manual releases, and a low risk profile makes it a relatively safe intervention (Bohunicky et al., 2024; Li et al., 2024). However, limitations include the physical effort required by therapists and the difficulty in standardizing of techniques for research or protocol development. Without a device to calibrate force or duration, manual therapy can be somewhat of an art, and outcomes might depend on the approach of the individual therapist. This variability contributes to mixed results seen in clinical studies. Nonetheless, MFR remains the foundation of many physical therapy & osteopathic treatments and provides a foundation upon which instrument-assisted and device-based methods have been developed (Ajimsha and Shenoy, 2019).

In summary, manual fascial release provides direct mechanical and sensory stimulation that may influence tissue mechanics and neurophysiological regulation. However, its effectiveness depends on operator skill, highlighting the need for more standardized and instrument-supported approaches.

## 4 Instrument-assisted myofascial release interventions: localized mechanical perturbation

Instrument-assisted fascial release techniques involve the use of external tools to aid the therapist in the mobilization of soft tissues and fascia. Its most prevalent form is known as (IASTM) (Seffrin et al., 2019). In IASTM, therapists utilize specially designed instruments to apply targeted pressure, scraping, or gliding strokes over the skin. These instruments are commonly made of stainless steel, plastic, or hard rubber. These tools can effectively detect and break down fascial adhesions or scar tissue (MacDonald et al., 2016; Bitra and Sudhan, 2019). Historically, instrument-assisted methods have been used since traditional East Asian medicine. The Chinese practice of gua sha (literal meaning “scraping petechiae”) used horn or jade tools to scrape skin until redness (Sha) appeared. It purportedly improves circulation and relieves pain (Wang et al., 2025). A similar practice existed in ancient Greek and Roman times with the strigil. Strigil is a curved metal tool used after baths to scrape the skin, which also has therapeutic effects. Modern IASTM techniques have refined these concepts using ergonomically shaped instruments and specific treatment protocols (Pianese and Bordoni, 2022).

### 4.1 Common IASTM systems and tools

Several IASTM techniques have gained popularity. These include Graston Technique^®^, ASTYM^®^ (Augmented Soft Tissue Mobilization), and Fascial Abrasion Technique. These systems differ slightly in the design of their tools and training methodology, but all operate on comparable principles. The Graston Technique uses a set of six stainless steel instruments with different curves and edges (some blunt, some sharper) to comb over tissues in various directions (McKivigan and Tulimero, 2020). Therapists detect vibration or gritty texture as the tool encounters tissue irregularities. These irregularities are presumed to be scar tissue or fascial adhesions. Repeated scraping in these areas is thought to cause a mild inflammatory response and mechanical breakdown of the adhesions. It initiates the remodeling phase, where new, more flexible tissue is laid down. In practical terms, IASTM may accelerate the resolution of excessive fibrosis and enhance the healing of tissue by controlled micro-injury followed by regeneration. It amplifies the concept of transverse friction massage, but uses tools to achieve a more uniform and deeper pressure (Bitra and Sudhan, 2019).

Apart from the scraping instruments, other tools and methods fall under instrument-assisted fascial release. Cupping therapy (myofascial decompression) is one such technique that has gained mainstream attention. Cupping involves placing suction cups on the skin to create negative pressure. This negative pressure lifts the underlying fascia and soft tissue (Jia et al., 2025). Traditional cupping uses heated glass cups (fire cupping) or mechanical pumps with plastic cups to draw the skin up. It decreases fascial adhesions by pulling layers apart, increases local blood flow, and relieves muscle tension. Modern sports cupping often involves moving silicone cups over lubricated skin (gliding cupping) to combine suction with movement, effectively performing a form of instrument myofascial release in reverse (lifting instead of pressing) (Deng et al., 2025). Some evidence indicates that cupping can reduce musculoskeletal pain and improve function. It sometimes performs on par with or better than standard care (Mohamed et al., 2023).

Another tool is the therapeutic massage roller stick (Tiger Tail or Stick roller). The patients or therapists can use these sticks to roll across muscle groups. It provides similar effects as foam roller but through a handheld instrument. These are simpler and don’t require body weight. It is thus effectively an instrument-assisted form of self-myofascial release (Medeiros et al., 2023). Fascial scraping tools in the beauty industry (like jade gua sha tools for the face) and percussion hammer devices (tapping tools used by some therapists) also exist, but are less common in general orthopedic rehabilitation (Hamp et al., 2023).

### 4.2 Application procedures and treatment parameters

During an IASTM session, the clinician applies a lubricant (non-greasy emollient) to the skin of patient to allow smooth tool movement. They then hold the instrument at a specific angle (30-60° to the skin) and perform strokes of variable pressure and direction over the target area. Superficial scanning strokes (diagnostic strokes) help to identify rough or grating sensations. Once adhesion is located, the therapist can do focused, deeper strokes (back-and-forth or cross-fiber) over that spot (Bitra and Sudhan, 2019). Patients often experience some discomfort, and treatment typically lasts a few minutes per area. It is common for IASTM to cause redness (erythema) and sometimes bruising (petechiae or ecchymosis) on the treated region (Cheatham et al., 2019). It is particularly found with techniques like gua sha or Graston, where the intent is to provoke tissue response. It is considered as a sign of increased circulation and an appropriate inflammatory reaction that stimulates healing. However, excessive bruising can be a downside for patient tolerance and may contraindicate frequent application. Typically, therapists limit IASTM to 2–3 sessions per week on a given area to allow recovery. Patients are often instructed to stretch and exercise to reinforce mobility gains after the session (Cheatham et al., 2016).

### 4.3 Functional and sensory outcomes

Common indications for instrument-assisted release include chronic tendinopathies (like lateral epicondylitis, Achilles tendinitis), postoperative or post-injury scar tissue, plantar fasciitis, iliotibial band syndrome, and general muscle/fascial tightness, especially in athletes (Cheatham et al., 2016). Therapists often incorporate IASTM in the subacute or chronic phase of injury rehabilitation to help remodel scar tissue once initial healing has taken place. In athletes, IASTM is used to address soft tissue restrictions that limit performance. IASTM has been reported to improve ankle dorsiflexion by treatment of the calf fascia in those with Achilles problems (Bhurchandi and Phansopkar, 2021). Likewise, Howitt et al. (2009) documented that treatment of triathletes’ acute tibialis posterior strain with the Graston Technique enabled a faster return to pain-free running. Collectively, such reports suggest that instrument-assisted methods can be effective in improving mobility and function in targeted issues (Howitt et al., 2009).

One notable advantage of instruments is reduced strain on the therapist. The tool concentrates force with less physical effort and can save the hands of therapists from fatigue or overuse injuries. Instruments with concave/convex shapes can mold around body contours and may reach areas where fingers cannot easily treat. They also provide magnified sensory feedback. The therapists report that the vibration of the tool in their hand helps them feel the texture of the tissue adhesions more clearly than with fingers alone (Tang et al., 2025). Additionally, IASTM sessions are often shorter than manual deep tissue work. A thorough instrument treatment of the region might take 5–10 minutes versus 15–20 minutes of comparable manual friction massage. It makes this technique time-efficient (MacDonald et al., 2016).

### 4.4 Risks, contraindications, and clinical limitations

Instrument-assisted techniques have a moderate evidence base. They appear effective for immediate improvements in flexibility or pain pressure thresholds. However, long-term functional improvements attributable solely to the instrument are harder to demonstrate. Despite these issues, IASTM continues to be widely used because it is relatively safe and often yields quick short-term results, which can motivate patients. Adverse effects are minor and mostly limited to transient soreness or superficial bruising. Proper training of the therapist or patient is important, as inappropriate technique (too aggressive, wrong angle) could cause tissue damage or unnecessary pain (Tang et al., 2025).

Overall, instrument-assisted techniques enable more targeted and consistent mechanical loading of fascial tissues while reducing therapist strain. These methods extend the principles of manual release through enhanced mechanical precision and reproducibility.

## 5 Device-mediated fascial modulation: self-applied and clinician-operated systems

These approaches include both core self-myofascial release methods and selected device-based interventions that influence myofascial tissue behavior through mechanical or acoustic stimulation. While not all device-based modalities constitute true myofascial release in the strict manual or instrument-assisted sense, they are included in this review where their clinical use, mechanical interaction, or proposed physiological mechanisms involve fascial tissue loading, fascial mechanoreceptor stimulation, or modulation of myofascial pain and mobility. This equipment ranges from simple foam rollers to advanced electro-mechanical instruments. These techniques often enable self-treatment by patients or enhance therapy through technology (Wiewelhove et al., 2019; Wuerfel et al., 2022; Michalak et al., 2024). The device-based approaches can be broadly classified into self-myofascial release (SMR) tools and clinician-applied devices.

### 5.1 Self-applied mechanical loading of fascial tissues

#### 5.1.1 Foam rollers and roller massagers

The most ubiquitous self-treatment device is a foam roller. Foam roller is firm, cylindrical roll with different densities used by individuals to apply body weight pressure to their muscles and fascia. Foam rolling technique is popular in fitness and rehabilitation communities as a method to perform myofascial release on oneself. It is often used as part of warm-up or cool-down routines (Zainuddin et al., 2025). Using a foam roller, a person can target large muscle groups (quadriceps, hamstrings, calves, iliotibial band, back muscles) by slowly rolling their body over the roller (Konrad et al., 2022). The pressure from the roller acts similarly to a massage or MFR from a therapist, but it is less targeted. The convenience and low cost of foam rollers have made them a staple in gyms and homes (Miller, 2024).

Past studies generally supports short-term and immediate benefits of foam rolling. Systematic reviews have found that self-myofascial release (SMR) with foam roll or roller massager can increase joint range of motion acutely without detrimental effects on the performance of muscle (Martínez-Aranda et al., 2024). Cheatham et al. (2015) concluded that a brief session (1–2 minutes per muscle group) of foam rolling can lead to immediate improvements in flexibility. Additionally, when it is used after intense exercise, foam rolling appears to reduce muscle soreness and improve recovery markers (Cheatham et al., 2015). Athletes who foam roll after workouts report less delayed onset muscle soreness (DOMS) and a quicker return of muscle performance when they are compared to those who don’t (Scudamore et al., 2021). It is also notable that foam rolling does not seem to cause harm. Different studies consistently show it does not negatively impact muscle function, even when done before exercise. This has led to recommendations that foam rolling is a useful adjunct to warm-ups and to recovery protocols (Adamczyk et al., 2020).

#### 5.1.2 Massage balls and therapy canes

Other self-myofascial release tools include massage balls (like lacrosse balls or spiky balls). These are excellent for pinpoint release (e.g., into the gluteal muscles or shoulder blade area) (Ferreira et al., 2022). Also, therapy canes or hooks are considered a self-myofascial release tool. These are plastic cane-shaped tools that allow a person to apply pressure to hard-to-reach spots such as between the shoulder blades. These devices enable individuals to treat trigger points and tight fascia on their own. It is done application of sustained pressure until release is felt. Many physical therapists and trainers recommend these as part of home exercise programs. They are recommended so patients can manage their myofascial restrictions daily in between therapy sessions (Ferreira et al., 2022; Miller, 2024).

From a mechanistic point of view, foam rolling works similarly to manual MFR by application of sustained pressure to fascia and muscle. It results in thixotropic changes and neurophysiological modulation of pain. The pressure of the body on the roller can produce self-induced discomfort, which may activate diffuse noxious inhibitory control. This reduces perceived pain after release. Foam rolling also enhances blood flow to the muscles. This increased blood flow can aid in recovery by flushing out metabolic byproducts (Michalak et al., 2024). Notably, foam rollers also come in various designs. Some have ridges or grids to supposedly target tissue more intensively. Some of them are vibrating foam rollers, which combine pressure with high-frequency vibration. The added vibration to foam rolling further increases the range of motion and pain tolerance. It is done by the provision of analgesic effect through vibration-induced sensory input. However, findings are mixed, and the added benefit of vibration is not conclusive (Alonso-Calvete et al., 2022).

### 5.2 Clinician-operated therapeutic devices

Apart from various self-treatment tools, there are a variety of electronic or mechanical devices that have been developed to assist with fascial release and soft tissue mobilization in clinical settings. These are as follows:

#### 5.2.1 Percussive and vibration therapy devices

One prominent category is percussive and vibration therapy devices. These handheld percussive massage guns (Theragun^®^, Hypervolt) have gained popularity in recent years among both therapists and consumers. These devices deliver rapid, repetitive strokes to the soft tissue (20–40 pulses per second) with an amplitude of a few millimeters. It essentially pummels the fascia and muscle. The sensation is a strong vibration and tapping combined. Percussive massage therapy is a related mechanical soft-tissue intervention commonly used for myofascial modulation that decreases muscle tightness, reduces soreness, and improves range of motion, with emerging evidence demonstrating measurable changes in fascial thickness and echo intensity in individuals with chronic low back pain (Yang et al., 2024). Users often glide the massage gun over major muscle groups for 1–2 minutes as a warm-up or recovery tool (Sams et al., 2023).

Another device-based approach in clinical use is therapeutic vibration via high-frequency oscillation devices. An example is the Rapid Release Therapy device, which vibrates at approximately 170 Hz with a very small amplitude. It is applied by gliding over the skin of areas with tissue restrictions (Lupowitz, 2022). Practitioners of this modality claim it can resonate with adhesions in fascia and scar tissue. It also helps to free stuck layers, akin to how an ultrasonic vibration might loosen particles. While rigorous evidence is sparse, anecdotal reports and pilot studies suggest that high-frequency vibration therapy can decrease pain and increase range of motion in conditions like neck stiffness and surgical scars. This is considered possibly by neurological mechanisms and enhanced circulation (Yilmaz Menek et al., 2024).

#### 5.2.2 Extracorporeal shockwave therapy

Although ESWT differs from traditional manual myofascial release in its use of acoustic energy, it is included here due to its clinical application in myofascial trigger points and fascial pain conditions. It is not classified as a primary myofascial release technique, but rather as a secondary fascial-targeted mechanical intervention due to its capacity to mechanically stimulate fascial tissues, modulate myofascial trigger points, and influence fascial-related pain and tissue remodeling processes. ESWT is another more powerful device-based intervention. It is originally used for breaking up kidney stones, but also adapted for musculoskeletal conditions. Shockwave units deliver focused acoustic waves into tissues. It causes a mechanical pressure effect and stimulates a healing response. ESWT has been used and studied mainly for tendinopathies and plantar fasciitis, but it is increasingly being applied to myofascial trigger points and chronic muscle/fascial pain areas. The shockwaves can disrupt calcifications and fibrous tissue and also induce neovascularization (new blood vessel formation), which promotes tissue regeneration and directly influence fascial fibroblast activity and extracellular matrix remodeling (Pirri et al., 2022; De la Corte-Rodríguez et al., 2023). For myofascial pain syndrome, evidence suggests shockwave therapy can significantly reduce pain and sensitivity of trigger points, at least in the short term (Avendaño-López et al., 2024). The proposed mechanisms include increased local blood flow, reduction of substance P (a pain neurotransmitter) in the tissue, and mechanical disruption of the trigger point’s taut band. Patients often require multiple sessions (3–5 treatments over several weeks). ESWT can be moderately uncomfortable during application but is usually well-tolerated and has the benefit of being quick (a few minutes of pulses per area) (Wuerfel et al., 2022).

#### 5.2.3 Adjunct physical modalities (ultrasound, RF, laser)

Other device modalities that can influence fascia include therapeutic ultrasound (providing deep heating to increase tissue extensibility), radiofrequency or diathermy (also heating tissue to allow stretch), and laser therapy (low-level laser might encourage tissue healing). These are generally adjuncts to facilitate fascial work (Ebadi et al., 2014; Jiménez-Sánchez et al., 2024). For instance, warming up fascia with ultrasound before manual stretching. They are not fascial release techniques per se, but they are preparatory or complementary modalities. Similarly, pneumatic compression devices (inflatable sleeves for lymphatic drainage) don’t directly release fascia but can reduce fluid congestion and possibly improve tissue mobility secondarily (Wiecha et al., 2021; Warneke et al., 2024).

### 5.3 Comparative characteristics of self-applied and clinician-applied modalities

Self-administered devices (foam rollers, massage guns used by the person) empower patients to manage their fascial health and can be done frequently as required. They rely on patient education for proper technique. For example, instructing a patient how to foam roll their IT band safely or how to use a massage gun on their quads without lingering too long on one spot (to avoid bruising) (Thomas, 2023). Clinician-operated devices like shockwave or certain vibrational tools are applied in the clinic for specific indications. They tend to be used when conventional manual or instrument methods aren’t able to resolve the issue. For instance, a therapist might decide to try shockwave on a chronic plantar fascia that hasn’t responded to manual massage and stretching. It is done in the hope of stimulating more robust healing response in that fascia (Manheim, 2024).

### 5.4 Technical, accessibility, and safety limitations

A limitation for device use is the cost and accessibility. Foam rollers and basic massage tools are cheap and widely available. In contrast, high-quality massage guns can be expensive, and shockwave machines are very costly and thus limited to clinics with those resources. Additionally, certain devices require expertise to use correctly (Kerautret et al., 2020). Shockwave requires knowledge of proper dosages and locations. Massage guns also come with contraindications such as not using over nerves, bony areas, or sensitive tissues (Ferreira et al., 2023). Thus, training and guidelines are important to prevent misuse. Generally, with reasonable use, these devices are safe.

Device-mediated interventions further expand the delivery of fascial loading through self-applied and technology-assisted modalities. These approaches improve accessibility and support integration of fascial release within broader rehabilitation strategies.

## 6 Comparative integration of fascial intervention modalities

### 6.1 Mechanistic basis for differential physiological effects across fascial release modalities

Although manual myofascial release (MFR), instrument-assisted soft tissue mobilization (IASTM), and device-mediated Myofascial release interventions share the overarching objective of improving fascial mobility and reducing pain. Yet, the physiological evidence suggests that their effects arise through partially distinct mechanobiological and neurophysiological pathways. These differences are primarily determined by variations in mechanical loading characteristics which influence extracellular matrix deformation, interstitial fluid behavior, mechanoreceptor activation, and fibroblast mechanotransduction. The mechanistic distinctions summarized in **Table 1** and the comparative characteristics presented in **Table 2** reflect these modality-specific physiological profiles.

**Table 2:** Comparative characteristics of manual, instrument-assisted, and device-based fascial release techniques.

Dimension	Manual fascial release (MFR)	Instrument-assisted soft tissue mobilization (IASTM)	Device-based techniques
Required Expertise	High clinician expertise and palpation skill	Moderate–high; instrument-specific training required	Variable; low for self-use, high for clinician-operated devices
Depth & Precision of Effect	Gentle–moderate; diffuse and adaptable	Moderate–deep; highly focal and targeted	Variable; superficial to deep (shockwave)
Primary Mechanisms	Sustained stretch and neurophysiological modulation	Compression and shear with localized remodeling	Mechanical loading, vibration, percussion, or acoustic stimulation
Typical Clinical Uses	Chronic pain, diffuse stiffness, centrally sensitized conditions	Localized adhesions, scars, tendinopathies	Warm-up, recovery, self-care, refractory chronic conditions
Efficacy (Pain & Function)	Moderate evidence for pain and mobility improvement	Comparable to MFR; faster effects in focal lesions	Strong short-term effects; strong evidence for shockwave in selected conditions
Duration of Effects	Hours–days; requires repeated sessions	Similar to MFR; typically delivered in series	Transient for self-tools; sustained for shockwave
Tolerance & Patient Preference	Generally high tolerance	Moderate tolerance; discomfort and bruising common	Variable; shockwave may be painful but brief
Side Effects/Risks	Minimal; transient soreness	Bruising, erythema, soreness; caution with fragile skin	Low risk with proper use; device-specific contraindications
Cost & Practicality	Clinic-based; time-intensive	Moderate equipment cost; clinic-based	Self-tools low cost; shockwave expensive and clinic-based
Best Context of Use	Acute–subacute phases, centrally mediated pain	Subacute–chronic focal tissue pathology	Self-care, athletic recovery, refractory chronic cases
Example Clinical Scenario	Fibromyalgia patient receiving whole-body MFR	Athlete with Achilles tendinopathy treated with IASTM	Runner using foam rolling; plantar fasciitis treated with ESWT
References	(Barnes and Rivera, 2022; Khan et al., 2022; Takenaka et al., 2023; Bohunicky et al., 2024; Li et al., 2024)	(Cheatham et al., 2019; McKivigan and Tulimero, 2020; Medeiros et al., 2023; Jia et al., 2025)	(Ebadi et al., 2014; Wuerfel et al., 2022; Jiménez-Sánchez et al., 2024)

### 6.2 Mechanical loading characteristics and extracellular matrix deformation

Manual MFR typically applies sustained, low-to-moderate load over extended durations. It allows gradual viscoelastic creep, stress relaxation, and thixotropic changes in the fascial extracellular matrix (Arguisuelas et al., 2017). This type of loading promotes redistribution of interstitial fluid and reduces hyaluronan viscosity. It facilitates improved glide between fascial layers without induction of significant structural disruption. Because the applied forces are distributed over broader surface areas and applied slowly, manual MFR primarily modifies tissue compliance and fluid dynamics rather than producing focal microstructural remodeling (Cowman et al., 2015).

In contrast, IASTM applies higher localized pressure and shear forces over smaller contact areas, It generate greater mechanical stress concentrations within targeted fascial regions. These higher stress gradients increase likelihood of disruption of fibrotic adhesions, abnormal collagen cross-links, and scar tissue. The resulting mechanical perturbation may initiate localized inflammatory and remodeling responses. These responses include fibroblast activation, collagen turnover, and neovascularization. This mechanism helps explain why instrument-assisted interventions are often particularly effective in conditions involving focal fibrosis, scar tissue, or chronic tendinopathy, where structural remodeling may be required to restore tissue mobility (Kim et al., 2017; Sandrey et al., 2020).

Device-mediated interventions exhibit modality-specific mechanical signatures. Foam rolling produces distributed compressive and shear loading that primarily alters viscoelastic properties and interstitial fluid distribution. It results in transient improvements in tissue compliance (Wilke et al., 2019). Percussive and vibration devices deliver high-frequency oscillatory mechanical input. They produce relatively small tissue displacements but strong sensory stimulation which favors neuromodulatory rather than structural effects. In contrast, high-energy acoustic interventions such as extracorporeal shockwave therapy deliver focused mechanical energy capable of penetration of deeper tissues and induce biological responses associated with tissue regeneration, angiogenesis, and nociceptor desensitization (Zhang et al., 2020; Rajfur et al., 2022).

These differences in mechanical loading profiles provide a physiological basis for the variable clinical effects and indications observed across fascial release modalities.

### 6.3 Differential activation of fascial mechanoreceptors and neuromodulatory pathways

Fascial tissues contain diverse mechanoreceptors, including Ruffini endings, Pacinian corpuscles, interstitial receptors, and free nerve endings. All of these possess distinct sensitivity to mechanical stimuli. The slow, sustained loading preferentially activates low-threshold mechanoreceptors such as Ruffini endings. These are associated with parasympathetic activation and inhibition of sympathetic tone. This mechanism contributes to reductions in muscle tone, generalized relaxation, and centrally mediated analgesic effects (Pratt, 2021; Sezik and Sezik, 2023).

In contrast, vibration-based and percussive interventions preferentially stimulate rapidly adapting mechanoreceptors such as Pacinian corpuscles and muscle spindle afferents. This stimulation alters proprioceptive signaling and spinal reflex excitability. It results in rapid neuromodulatory effects on muscle tone and pain perception. These effects help explain the rapid but often transient improvements in range of motion and pain sensitivity observed following vibration-based interventions (Yilmaz Menek et al., 2024).

IASTM and friction-based techniques activate both mechanoreceptors and nociceptors due to higher shear forces and localized mechanical stress. The resulting nociceptive input may engage descending inhibitory pathways like diffuse noxious inhibitory control mechanisms and contributes to pain modulation. Additionally, localized inflammatory signaling induced by mechanical stress may facilitate remodeling of tissue through mechanobiological pathways. These modality-specific neural activation patterns help explain why manual techniques often produce broader neuromodulatory effects, while instrument-assisted and high-energy interventions may produce more localized structural and sensory changes (Tobaldini et al., 2019; Lowery et al., 2025).

### 6.4 Effects on interstitial fluid dynamics and fascial glide

Fascial mobility depends critically on interstitial fluid dynamics and extracellular matrix hydration. Sustained manual loading facilitates gradual fluid redistribution and reduces hyaluronan viscosity and improves the lubrication and sliding of fascial layers. These effects are particularly relevant in conditions which are characterized by fascial densification, where increased extracellular matrix viscosity impairs normal tissue mobility (Streďanská et al., 2025).

Instrument-assisted and shockwave interventions may produce more pronounced vascular and fluid responses due to higher mechanical energy transfer. These effects can enhance local circulation, improve metabolic exchange, and facilitate tissue remodeling in chronically stiff or fibrotic tissues (Miners and Bougie, 2011). In contrast, self-administered techniques like foam rolling primarily enhance transient fluid redistribution and compliance. This contribute to short-term improvements in mobility and pain sensitivity (Vatovec et al., 2024). The magnitude and persistence of these fluid-related effects are strongly influenced by intervention intensity, duration, and frequency. It provides physiological explanation for variability in treatment outcomes across different modalities and dosing protocols.

### 6.5 Cellular mechanotransduction and structural remodeling responses

Mechanical loading influences fibroblast behavior through mechanotransduction processes that regulate extracellular matrix turnover and tissue remodeling. Sustained, low-intensity loading primarily alters fibroblast cytoskeletal tension and extracellular matrix organization without induction of significant inflammatory responses. These effects contribute to gradual normalization of tissue compliance and improved fascial glide (Kodama et al., 2023).

Higher-intensity mechanical stimuli, such as those applied during IASTM or shockwave therapy, may induce localized inflammatory signaling and activate fibroblast proliferation, collagen synthesis, and angiogenic pathways. These cellular responses contribute to structural remodeling of fibrotic or scarred tissues and may explain the greater effectiveness of these interventions in chronic conditions involving structural fascial alterations. However, higher mechanical intensity may also increase discomfort, tissue irritation, or patient intolerance, highlighting the importance of appropriate dosing and clinical judgment in selecting intervention parameters (Candeniz et al., 2023; Hwang et al., 2025). These cellular responses are mediated through mechanotransduction pathways, where mechanical loading influences fibroblast activity, cytokine expression, and extracellular matrix remodeling, contributing to restoration of normal fascial viscoelastic properties (Ide et al., 2026).

Although manual myofascial release and instrument-assisted soft tissue mobilization share similar therapeutic objectives and mechanobiological principles, important differences exist in their mechanical delivery and clinical effects. Manual myofascial release typically applies sustained, distributed pressure over broader tissue regions, favoring gradual viscoelastic deformation, fluid redistribution, and neuromodulatory effects mediated through mechanoreceptor stimulation (Manheim, 2024; Shults and Fritsch, 2025). In contrast, instrument-assisted techniques enable more localized and concentrated mechanical loading, which may facilitate targeted disruption of fibrotic adhesions and more pronounced mechanical perturbation of restricted fascial regions (Seffrin et al., 2019). Clinically, both approaches have demonstrated improvements in pain, range of motion, and functional outcomes in chronic musculoskeletal conditions. Manual techniques may be particularly effective for generalized fascial stiffness and neuromodulatory effects, whereas instrument-assisted techniques may provide advantages in treating focal fibrosis, scar tissue, or localized fascial restrictions (Cabrera-Martos et al., 2022; Tehrani et al., 2022; Bingölbali et al., 2024). However, current evidence does not consistently demonstrate clear superiority of one modality over the other, and their complementary use within multimodal rehabilitation programs may provide the greatest clinical benefit.

While these approaches share overlapping therapeutic goals, their physiological effects differ depending on mechanical loading characteristics, tissue interaction profiles, and neurosensory engagement. Understanding these mechanistic differences is essential for explaining modality-specific effects and guiding targeted clinical application.

## 7 Clinical implications and therapeutic effects in musculoskeletal conditions

Within the context of integrative physiology, myofascial release interventions should be interpreted less as discrete therapeutic entities and more as structured mechanical and sensory inputs capable of modulating interconnected myofascial, neural, and autonomic systems. This perspective emphasizes physiological responsiveness and system-level integration over assumptions of consistent, modality-specific structural remodeling of fascial tissue. The integration of myofascial release and fascial-targeted mechanical interventions into clinical practice provides clinicians with a variety of tools. These tools help address musculoskeletal pain and movement dysfunctions that do not respond completely to conventional interventions (El-Tallawy et al., 2021). It is due to the rise in fascia awareness as a contributing factor to movement, force transmission, and pain perception that enhances treatment options in the context of rehabilitation and performance (Kodama et al., 2023).

Within clinical practice, the therapeutic effects of myofascial release and fascial-targeted mechanical interventions have been reported across a range of musculoskeletal conditions, with outcomes varying according to anatomical region, underlying pathology, and intervention approach. In patients with chronic low back pain, interventions targeting the thoracolumbar fascia have been associated with reductions in pain intensity, improvements in lumbar mobility, and enhanced functional capacity, suggesting that modification of fascial stiffness and interlayer glide may contribute to symptom relief (Devantéry et al., 2023; Vining et al., 2022). Similarly, in cervical and upper trapezius regions, myofascial release has demonstrated beneficial effects in individuals with chronic neck pain, including increased range of motion, reduced pain sensitivity, and improvements in functional disability measures, which may reflect both mechanical and neurophysiological modulation (Cabrera-Martos et al., 2022; Overmann et al., 2024).

In lower extremity disorders, particularly plantar fasciitis and Achilles-related conditions, myofascial release and instrument-assisted techniques applied to the plantar fascia and posterior chain have been associated with improvements in pain, walking tolerance, and ankle mobility. These effects may be related to enhanced fascial compliance, reduced localized stiffness, and improved load distribution (Lipa et al., 2022). In tendinopathies and regional overuse conditions such as lateral epicondylitis and iliotibial band syndrome, fascial-targeted interventions have demonstrated improvements in pain and joint mobility, particularly where fascial adhesions or fibrotic changes are suspected to contribute to movement restriction (Kannabiran et al., 2017; Miccio et al., 2021).

Myofascial pain syndrome represents another important clinical context in which fascial-targeted interventions have demonstrated therapeutic value. Techniques including manual myofascial release, instrument-assisted mobilization, and selected device-based interventions have been associated with reductions in trigger point sensitivity, decreased muscle stiffness, and improvements in functional performance. Across these diverse conditions, commonly reported outcome indicators include pain intensity scales, range of motion assessments, pressure pain thresholds, and functional disability measures (Tehrani et al., 2022; Bingölbali et al., 2024).

Collectively, these findings support the role of myofascial release as a region-specific adjunct within comprehensive rehabilitation strategies. However, variability in treatment protocols, patient populations, and outcome measures highlights the importance of individualized clinical reasoning and integration with active rehabilitation approaches to achieve sustained functional improvement. A summary of clinical application parameters, therapeutic context, and evidence patterns for myofascial release and fascial-targeted mechanical interventions is provided in **Table 3**. **Table 3** also summarizes typical clinical indications and anatomical regions for each intervention modality, reflecting the heterogeneity of treatment applications across musculoskeletal conditions. These summaries are derived from synthesis of multiple randomized trials, systematic reviews, and clinical studies across various musculoskeletal conditions, including low back pain, neck pain, plantar fasciitis, tendinopathies, and myofascial pain syndrome.

**Table 3:** Clinical application parameters and evidence context of myofascial release and fascial-targeted mechanical interventions in musculoskeletal rehabilitation.

Intervention category	Primary mode of force application	Level of clinician involvement	Typical session duration	Frequency	Common treatment course	Patient autonomy	Typical clinical phase	Typical clinical indications and anatomical regions	References
Manual Fascial Release (MFR)	Sustained compression, traction, slow stretch	High	2–10 min	1–2/week	4–8 weeks	Low	Acute–chronic	Chronic low back pain (thoracolumbar fascia), neck pain (cervical fascia), myofascial pain syndrome (regional fascial restrictions)	(Arguisuelas et al., 2017; Bandl et al., 2022; Ide et al., 2026)
Direct Deep Fascial Techniques	Deep compression and shear	High	5–20 min	1/week	6–10 sessions	Low	Subacute–chronic	Chronic fascial adhesions, postural dysfunction, global fascial restrictions (thoracolumbar fascia, lower extremity fascial chains)	(Shamsi et al., 2024; Ide et al., 2026)
Active Fascial Techniques (ART)	Compression + active/passive movement	High	1–5 min	1–3/week	3–6 weeks	Low	Subacute–chronic	Tendinopathies, muscle–fascial adhesions (Achilles tendon, upper trapezius, forearm extensors)	(VK and Kumar, 2019; Barnes and Rivera, 2022)
IASTM	Focused compression & shear via tools	Moderate–high	3–10 min	2–3/week	4–6 weeks	Low	Subacute–chronic	Tendinopathies (Achilles, lateral epicondylitis), plantar fasciitis, postoperative scar tissue, localized fascial fibrosis	(MacDonald et al., 2016; Rowlett et al., 2019; Lin et al., 2025)
Fascial Decompression (Cupping)	Negative pressure traction	Moderate	5–15 min	1–2/week	3–6 weeks	Low–moderate	Subacute–chronic	Chronic myofascial pain, neck and shoulder pain, thoracolumbar fascial stiffness	(Mohamed et al., 2023; Deng et al., 2025; Jia et al., 2025)
Self-Myofascial Release (SMR)	Body-weight compression, rolling shear	Low	30–120 s/muscle	Daily	Ongoing	High	Acute, recovery, chronic	Generalized fascial stiffness, post-exercise recovery, lower extremity fascial restrictions (quadriceps, calves, plantar fascia)	(Ferreira et al., 2022; Martínez-Aranda et al., 2024)
Percussive/Vibration Devices	Rapid oscillatory/percussive loading	Low–moderate	30–90 s/muscle	Daily/alternate	Ongoing	Moderate–high	Warm-up, recovery, chronic	Muscle stiffness, sports recovery, trigger point-related fascial restrictions (upper trapezius, quadriceps, calf fascia)	(Lupowitz, 2022; Sams et al., 2023)
Shockwave Therapy (ESWT)	Acoustic pressure waves	High	2–5 min	1/week	3–5 sessions	None	Chronic refractory	Chronic plantar fasciitis, myofascial trigger points, chronic tendinopathies (Achilles, shoulder, elbow)	(Ryskalin et al., 2022; De la Corte-Rodríguez et al., 2023; Avendaño-López et al., 2024)

MFR, myofascial release; IASTM, instrument-assisted soft tissue mobilization; ART, active release technique; ESWT, extracorporeal shockwave therapy. Treatment parameters represent commonly reported clinical ranges derived from multiple randomized controlled trials, systematic reviews, and clinical studies across musculoskeletal conditions. These values are intended to reflect general clinical practice patterns rather than prescriptive treatment protocols.

## 8 Current evidence, methodological limitations, and knowledge gaps

Although there is an increase in clinical use and publication volume, the evidence base for fascial release remains limited by considerable methodological and conceptual constraints. It involves highly variable interventions (manual MFR, IASTM, foam rolling, cupping, vibration devices), performed with inconsistent forces, duration, equipment, and areas of treatment. Similarly, the results are also diverse (pain, ROM, function, quality of life). This heterogeneity limits comparability and weakens meta-analytic conclusions. In addition, placebo and expectancy effects are difficult to control because blinding is rarely reliable for hands-on or mark-producing techniques (bruising after IASTM/cupping), and therapist enthusiasm or contextual effects may inflate outcomes. The majority of the reports focus on the short-term or immediate outcomes with minimal long-term follow-ups. It creates questions in terms of longevity, requirements of maintenance, and whether the effects of the benefits are structural adaptation or temporary neuro-modulation. A further gap is the lack of direct fascia-specific measurement.

In recent years, advances in imaging and biomechanical assessment have provided new opportunities to evaluate fascial structure and mechanical behavior *in vivo*. Ultrasound imaging has been widely used to assess fascial thickness, morphology, and sliding between tissue layers, while magnetic resonance imaging (MRI) offers high-resolution visualization of fascial architecture and pathological alterations. Ultrasound elastography techniques, including shear wave elastography, allow quantification of fascial stiffness and viscoelastic properties, providing objective measures of tissue mechanical behavior. In addition, handheld biomechanical devices such as myotonometry instruments can assess tissue stiffness, elasticity, and mechanical responsiveness at clinically accessible sites. Importantly, emerging evidence suggests that these structural and biomechanical parameters may correlate with clinical indicators such as pain intensity, range of motion, and functional limitations. Integrating objective fascial assessment with clinical outcome measures may improve understanding of treatment mechanisms, enhance patient stratification, and support more targeted application of myofascial release interventions.

The changes in the pain or ROM do not verify the alteration of fascial stiffness, hydration, adhesion, or inter-layer glide. Lastly, the quality of the studies is rather moderate (small groups, the risk of bias, and publication bias). The interventions are frequently confounded by concurrent therapies (exercise/education). Also, mechanisms remain difficult to isolate because fascia operates within the myofascial–neural system. Also, key populations (older adults, pediatrics, connective tissue disorders, and metabolic conditions) are underrepresented. It collectively requires cautious interpretation and avoidance of overpromising. A synthesis of current evidence strength and research gaps across myofascial release and fascial-targeted mechanical interventions is provided in **Table 4**.

**Table 4:** Summary of evidence strength for common fascial release techniques based on randomized controlled trials and systematic reviews, highlighting short- and long-term clinical efficacy and research gaps.

Technique	No. of studies (design)	Evidence strength	Short-term efficacy	Long-term efficacy	Key gaps	References
Manual MFR	Systematic Reviews (various pain conditions)	Weak/inconclusive	Some small pain/ROM improvements reported	Not established (few studies >4–8 wk)	Heterogeneous methods; small sample sizes; no standardized dosing	(Ajimsha and Shenoy, 2019)
IASTM (Instrument)	13 RCTs (Graston/ASTYM, etc.)	Moderate (positive effect sizes)	↑ ROM in healthy; ↓ pain and ↑ function in injured patients	Short follow-ups (usually <1 mo)	Few large trials; variable tools/protocols	(Seffrin et al., 2019)
Shockwave (ESWT)	Many RCTs/meta-analyses (19 RCTs in tendinopathies)	Strong in select conditions (1+ level in calcific tendon/epicondylitis); overall moderate	Significant pain relief (e.g. VAS ∼−1.49) and functional gains	Benefits often last 3–12 mo	Protocol heterogeneity (energy, frequency); optimal dosing unclear	(De la Corte-Rodríguez et al., 2023)
Self-MFR (foam rolling)	21 RCTs (foam rollers/massage tools)	Moderate	Small ↑ ROM (~4%); modest ↓ post-exercise soreness; no loss of strength	No long-term data	Variable timing/intensity; lack of placebo/sham controls	(Wiewelhove et al., 2019)
Cupping Therapy	10 RCTs (chronic MSK pain)	Moderate for pain (SMD∼–1.17); weak for function	Immediate pain relief (SMD≈–1.17); no disability change	Not studied	High heterogeneity (cupping type, controls); blinding difficult	(Jia et al., 2025)
Vibration/Percussive (guns)	11 trials (massage guns/WBV)	Limited/moderate	↑ ROM/flexibility in target muscles; may reduce stiffness; no strength gain	Not evaluated	Few RCTs; diverse devices/settings; short-term protocols	(Ferreira et al., 2023)

## 9 Future directions in fascial physiology research

Future progress depends on reinforcement of both clinical trials and mechanistic science. The areas of priority are bigger, well-controlled studies with standardized and transparently reported protocols, better sham designs, and follow-ups. Simultaneously, mechanistic studies should elucidate biomechanotransduction programs, inflammatory/remodeling and central pain-modulatory mechanisms. Development and clinical uptake of objective assessment tools (e.g., ultrasound elastography or portable stiffness metrics) could enable quantification of fascial properties, better patient stratification, and responsive monitoring. Innovation is also likely in integrative approaches, along with patient-specific tailoring and interdisciplinary education, to standardize competency across professions. As evidence matures, clearer clinical guidelines and more precise indications should emerge. The applications should be expanded where appropriate while reducing reliance on anecdote and market-driven claims.

## 10 Conclusion

Myofascial release techniques constitute a primary group of mechanically applied interventions targeting fascial tissues, while selected fascial-targeted mechanical interventions extend these principles through device-assisted or self-administered modalities. Within this framework, primary myofascial release techniques include manual and instrument-assisted interventions that directly target fascial tissues, while selected device-based modalities are included where their mechanisms involve fascial mechanical loading or modulation of fascial-related neuromechanical function. When applied judiciously, these approaches may help address stiffness, pain sensitivity, and mobility restrictions that persist despite conventional stretching, particularly where altered fascial glide or adhesions are clinically suspected. From an integrative physiological perspective, the effects of fascial release are best interpreted as arising from the interaction of mechanical tissue loading, sensory receptor stimulation, and neurophysiological modulation rather than from consistent, modality-specific structural remodeling of fascial tissue. Consequently, symptom relief and transient mobility gains achieved through Myofascial release interventions should be reinforced through active rehabilitation strategies, including therapeutic exercise, motor control retraining, and progressive functional loading, to support durable clinical outcomes and reduce recurrence. At the same time, the evidence base remains limited by intervention heterogeneity, challenges in blinding (particularly for mark-producing techniques), predominantly short-term outcome horizons, and scarce fascia-specific measurement—factors that complicate attribution of benefit to structural fascial change versus transient neuromodulation. Future progress should prioritize standardized, transparently reported protocols; improved sham designs; longer follow-up; and objective tools (e.g., elastography) to quantify fascial properties and support patient stratification.
